# Screening for cardiovascular risk factors in adults with serious mental illness: a review of the evidence

**DOI:** 10.1186/s12888-015-0416-y

**Published:** 2015-03-21

**Authors:** Julia B Baller, Emma E McGinty, Susan T Azrin, Denise Juliano-Bult, Gail L Daumit

**Affiliations:** 1 Johns Hopkins Bloomberg School of Public Health, 624 N Broadway St., Room 405, Baltimore, MD 21205 USA; 2 National Institute of Mental Health, 6001 Executive Boulevard, Room 7145 MSC 9631, Rockville, MD 20852 USA; 3 Johns Hopkins School of Medicine, 2024 E Monument St, Suite 2-620, Baltimore, MD 21205 USA

## Abstract

**Background:**

Adults with serious mental illness have a mortality rate two to three times higher than the overall US population, much of which is due to somatic conditions, especially cardiovascular disease. Given the disproportionately high prevalence of cardiovascular risk factors in the population with SMI, screening for these conditions is an important first step for timely diagnosis and appropriate treatment. This comprehensive literature review summarizes screening rates for cardiovascular risk factors in the population with serious mental illness.

**Methods:**

Relevant articles published between 2000 and 2013 were identified using the EMBASE, PsychInfo, PubMed, SCOPUS and Web of Science databases. We reviewed 10 studies measuring screening rates for obesity, diabetes, dyslipidemia, and hypertension in the population with serious mental illness. Two reviewers independently extracted information on screening rates, study population, and study setting.

**Results:**

Rates of screening varied considerably by time period, study population, and data source for all medical conditions. For example, rates of lipid testing for antipsychotic users ranged from 6% to 85%. For some conditions, rates of screening were consistently high. For example, screening rates for hypertension ranged from 79% - 88%.

**Conclusions:**

There is considerable variation in screening of cardiovascular risk factors in the population with serious mental illness, with significant need for improvement in some study populations and settings. Implementation of standard screening protocols triggered by diagnosis of serious mental illness or antipsychotic use may be promising avenues for ensuring timely diagnosis and treatment of cardiovascular risk factors in this population.

**Electronic supplementary material:**

The online version of this article (doi:10.1186/s12888-015-0416-y) contains supplementary material, which is available to authorized users.

## Background

Persons with serious mental illnesses (SMI) such as schizophrenia and bipolar disorder have a mortality rate two to three times higher than the overall United States (US) population [1,2]. Much of this premature mortality is due to comorbid somatic conditions, particularly cardiovascular disease [3-6]. Those with SMI experience a high burden of cardiovascular risk factors, including obesity, diabetes, dyslipidemia, and hypertension [7,8]. Elevated rates of cardiovascular risk factors in this group are driven by low rates of physical activity, [9] poor diet, [10] high rates of smoking, [11] and the obesogenic effects of commonly prescribed antipsychotic medications [12], which often cause weight gain and alter glucose metabolism.

Given the disproportionately high prevalence of cardiovascular risk factors in the population with SMI, screening for these conditions is an important first step for timely diagnosis and appropriate treatment. The United States Preventive Services Task Force (USPSTF), a federal panel of experts in prevention and evidence-based medicine, makes recommendations about systematic screening in primary care for the overall US population. For example, all Americans with sustained blood pressure greater than 135/80 mmHg are recommended to be screened for diabetes mellitus [13]. These recommendations are based on strong bodies of research evidence demonstrating that the preventive service leads to improved health outcomes, which is often the result of diagnosis and effective treatment following screening [13].

In 2004, the American Diabetes Association, American Psychiatric Association, American Association of Clinical Edocrinologists, and North American Association for the Study of Obesity (hereafter referred to as the “ADA/APA consensus panel”) [14] released guidelines for screening and monitoring of somatic conditions among antipsychotic users, many of whom have SMI. The ADA/APA consensus panel guidelines recommend more frequent screening for and monitoring of cardiovascular risk factors among antipsychotic users than the USPSTF guidelines recommend for the overall US population. Unlike the USPTF guidelines, which are based on robust bodies of research evidence demonstrating a link between guideline-concordant screening and improved health outcomes, the ADA/APA guidelines are based on physician consensus.

Although research suggests that the population of adults with SMI experiences a disproportionately high burden of treatable cardiovascular risk factors [15,16] and that systematic screening and treatment may improve health outcomes, [17,18] to our knowledge no comprehensive review of the literature exists regarding rates of screening for cardiovascular risk factors in the population with SMI. To fill this gap, we reviewed the peer-reviewed literature published between January 2000 and December 2013 to summarize measures of screening for cardiovascular risk factors in the population with SMI. We abstracted measures related to screening for cardiovascular risk factors in various study samples of persons with SMI.

## Methods

Development of our review followed a modified PRISMA (Preferred Reporting Items for Systematic Reviews and Meta-Analyses) framework, an evidence-based set of items for reporting reviews. The goal of our review was to summarize descriptive measures of screening rates for cardiovascular risk factors in the adult population with SMI. We used a structured review protocol (Additional file 1) to abstract measures of screening for cardiovascular risk factors in study populations with SMI. A key limitation to our modified approach was our inability to systematically assess the quality of the included studies. Because we excluded randomized clinical trials and other intervention studies, we did assess the risk of bias for individual studies as is typically done in systematic reviews of clinical trials. These bias assessments focus on indicators of internal validity, which are not relevant for purely descriptive studies. We did include observational studies that assessed screening rates at multiple time points, for example before and after the release of relevant clinical guidelines. For these studies, we abstracted and presented screening time period-specific screening rates, e.g. rates for both 2002–2003 and 2005–2007.

In our final review, we abstracted measures of screening for five cardiovascular risk factors and one risk behavior shown by prior research to be highly prevalent among the population with SMI: [16,19-21] (1) overweight and obesity; (2) diabetes mellitus; (3) dyslipidemia; (4) hypertension; and (5) tobacco use. We also searched for studies measuring rates of screening for tobacco use among persons with SMI. We searched EMBASE, PsychInfo, PubMed, SCOPUS and Web of Science for studies published from January 2000 through December 2013 that measured screening rates for the conditions listed above in the population with SMI. Full search strategies are included in Additional file 2. For the purposes of this review, we defined SMI as schizophrenia or bipolar disorder. Studies that included persons with other diagnoses, such as major depression or post-traumatic stress disorder (PTSD) were included if the study also included participants with schizophrenia or bipolar disorder. Studies comprised entirely of persons with major depression, PTSD, or other conditions were excluded.

Studies were included in our comprehensive review if (1) they were published between January 2000 and December 2013; (2) they were published in English; (3) they included participants aged 18 years or older; (4) they measured rate of screening for cardiovascular risk in a study population with SMI; (5) the study sample included 50 or more participants; (6) the study took place in the US; (7) the study population included persons with schizophrenia or bipolar disorder; and (8) the study was observational. As the goal of this review was to assess rates of screening for cardiovascular risk factors in usual-care scenarios for persons with SMI, randomized clinical trials and other intervention studies designed to improve screening rates were excluded from this manuscript and included in a separate review of intervention studies by the same authors.

Two reviewers abstracted measures of screening for cardiovascular risk factors from the articles (for example, glucose testing among persons with schizophrenia to screen for diabetes mellitus). The reviewers abstracted data about the study design and population, including year(s) the data was collected, number of study participants, the study population’s SMI diagnoses and antipsychotic use, and study setting characteristics including type of setting (e.g. inpatient, outpatient, non-clinical community), geographic location (e.g. national, state), and other population characteristics (e.g. veterans or Medicaid beneficiaries). A copy of the abstraction tool is included in Additional file 1.

Using data abstracted from the studies included in our review, we compiled all available measures and created tables including information on the measurement indicator, data source, study population, year of data, and measures of screening in the population with SMI, including information about the time window for each screening rate (e.g. annual screening, screening in the 30 days after antipsychotic medication initiation, etc.). We then compared rates of screening for cardiovascular risk factors in the studies reviewed to national quality of care guidelines. When national estimates were available, we compared screening rates in the population with SMI to screening rates in the overall US population. As no human subjects were involved in this research, informed consent for participation was not obtained and institutional review board review of the study was not required.

## Results

**Figure 1:**
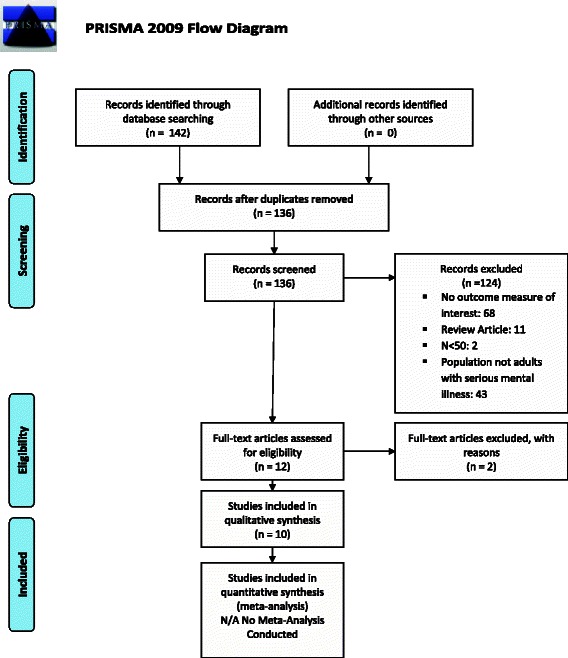
**PRISMA flow diagram, included studies.**

Our search yielded a total of 136 studies (Figure 1). One hundred twenty-six studies were excluded for failure to meet inclusion criteria; in this category, most studies were excluded for not having an outcome measure of interest. A total of 10 studies were included in our review. Included studies measured screening rates for overweight and obesity, diabetes mellitus, dyslipidemia, and hypertension. While we also searched for studies measuring rates of screening for tobacco use in the population with SMI, no studies measuring this outcome that met our inclusion criteria were identified.

### Demographic characteristics of the study populations reviewed

The demographic characteristics of the study populations included in our review are shown in Table 1. Populations varied by sex, race, and SMI diagnoses. Overall, most studies included adults between the ages of 18 and 65. Six of the 10 included studies had populations comprised 100% of persons with schizophrenia or bipolar disorder. Six study populations were made up entirely of antipsychotic medication users, and the remaining four studies did not report the proportion of participants using antipsychotics.

**Table 1:** **Study population characteristics**

Study	Age range	Mean age	Sex	Race	Proportion of sample with schizophrenia and bipolar disorder	Antipsychotic use
Kilbourne, 2011 [22]	18-65	55	9% female	24% black,	100% Schizophrenia	100%
				76% other		
Morrato, 2008 [25]	All ages	Not reported	50% female	58% white,	100% Schizophrenia^1^	100%
				42% other		
Moeller, 2011 [26]	18-64	44	49% female	83% white,	100% Schizophrenia	100%
				17% other		
Morrato, 2009 [27]	20-88	Not reported	50% female	Not reported	3.4% Schizophrenia, 49.3% Mood disorders, including bipolar disorder	100%
Kaplowitz, 2006 [30]	20+	67	4% female	Not reported	51.0% Mood disorders, including bipolar disorder	Not reported
Weissman, 2006 [31]	18-65	50	6% female	Not reported	100% Schizophrenia	100%
Barnett, 2010 [32]	21-63	45	59% female	43% white,	Not reported	100%
				14% black,		
				43% other		
Kilbourne, 2008 [34]	18+	67	3% female	8% black,	100% Schizophrenia, bipolar disorder, or other psychosis^2^	Not reported
				92% other		
Folsom, 2002 [35]	45+	51	47% female	70% white,	100% Schizophrenia^3^	Not reported
				15% black,		
				15% other		
Dickerson 2003 [38]	18-65	44	53% female	56% white,	50% Schizophrenia, 25% Bipolar disorder	Not reported
				35.5% black,		
				8.5% other		

^1^The full study population also included individuals with other or no mental health diagnoses, but all screening measures abstracted for this review were assessed in the study population with schizophrenia only.

^2^The full study population also included individuals with depression and individuals with no psychiatric diagnoses, but all screening measures abstracted for this review were assessed in the study population with schizophrenia, bipolar disorder, or other psychoses only.

^3^The full study population also included individuals with depression, but all screening measures abstracted for this review were assessed in the study population with schizophrenia only.

### Overweight and obesity (Panel 1)

The USPSTF recommends screening all adults for obesity [13]. For antipsychotic users with SMI, the ADA/APA consensus panel recommends screening for overweight and obesity at time of antipsychotic initiation, four, eight, and 12 weeks after initiation of a new antipsychotic medication, and four times per year thereafter [14].

In our review, we found one study that measured overweight and obesity screening in the population with SMI. One hundred percent of this population was taking antipsychotics. The screening and monitoring rate for overweight and obesity was relatively high (76%) (Table 2) [22]. This study consisted of a 2007 veteran population. Participants were considered to have been screened for obesity if their BMI was measured twice in 2007. In comparison to the population with SMI, 50% of visits to office-based physicians in the US had the height and weight measurements necessary to screen for overweight and obesity using Body Mass Index (BMI) in 2005–2006, [23] and in 2010, 71% of office-based physician visits included weight assessments (Table 3) [24].

**Table 2:** **Measures of screening for cardiovascular risk factors in persons with serious mental illness, 2000-2013**

Study population	Main finding in words	References
**Panel 1: Screening for Overweight and Obesity**		
**Body Mass Index**		
**N:** 40600 **| % of study sample with schizophrenia or bipolar disorder:** 100% **| Percent antipsychotic users:** 100% **| Study population:** Outpatient Veterans participating in the National Psychosis Registry, **| Screening year(s):** 2007 **| Time window for ascertaining screening rate:** Bi-annual **| Data source:** Administrative claims	76% of patients had Body Mass Index (BMI) assessed in 2007	Kilbourne, 2011 [22]
**Panel 2: Screening for Diabetes Mellitus**		
**Glucose screening**		
**N:** 55436 **| % of study sample with schizophrenia or bipolar disorder:** 100% **| Percent antipsychotic users:** 100% **| Study population:** Inpatient and Outpatient Medicaid beneficiaries from California, Oregon, Tennessee and Utah **| Screening year(s):** 1998–2003 **| Time window for ascertaining screening rate:** 14 days prior-28 days after second generation antipsychotic initiation **| Data source:** Administrative claims	18% of those without schizophrenia and 23% of those with schizophrenia had a glucose test during 1998-2003	Morrato, 2008 [25]
	Compared to those without schizophrenia, those with schizophrenia had 1.5 times greater odds of having a glucose test during 1998–2003 (p < .05)	
**N:** 18876 **| % of study sample with schizophrenia or bipolar disorder:** 3.4% schizophrenia and 49.3% mood disorders, including bipolar disorder **| Percent antipsychotic users:** 100% **| Study population:** Inpatient and outpatient commercial health insurance beneficiaries **| Screening year(s):** 2001–2006 **| Time window for ascertaining screening rate:** 30 days prior-30 days after second generation antipsychotic initiation **| Data source:** Administrative claims	The mean glucose testing rate for antipsychotic-treated patients was 23% during 2001-2006	Morrato, 2009 [27]
**N:** 2204 **| % of study sample with schizophrenia or bipolar disorder:** 100% **| Percent antipsychotic users:** 100% **| Study population:** Inpatient and outpatient Kansas Medicaid beneficiaries **| Screening year(s):** 2003 **| Time window for ascertaining screening rate:** Annual **| Data source:** Administrative claims	23% of patients had a glucose test during 2002-2003	Moeller, 2011 [26]
	29% of patients with dyslipidemia had a glucose test during 2002-2003	
**N:** 1646**| % of study sample with schizophrenia or bipolar disorder:** 100% **| Percent antipsychotic users:** 100% **| Study population:** Inpatient and outpatient Kansas Medicaid beneficiaries**| Screening year(s):** 2005-2007**| Time window for ascertaining screening rate:** Annual **| Data source:** Administrative claims	75% of patients had a glucose test during 2005-2007	
	65% of patients with dyslipidemia had a glucose test during 2005-2007	
**N:** 6601 **| % of study sample with schizophrenia or bipolar disorder:** Not reported **| Percent antipsychotic users:** 100% **| Study population:** Inpatient and outpatient California Medicaid recipients **| Screening year(s):** 2004-2005**| Time window for ascertaining screening rate:** Six months prior to-6 months after second generation antipsychotic initiation**| Data source:** Administrative claims	24% of patients had a glucose test in the six months prior to antipsychotic initiation during 2004-2005	Barnett, 2010 [32]
	28% of patients had a glucose test in the six months after antipsychotic initiation during 2004-2005	
**N:** 40600 **| % of study sample with schizophrenia or bipolar disorder:** 100% **| Percent antipsychotic users:** 100% **| Study population:** Outpatient Veterans participating in the National Psychosis Registry **| Screening year(s):** 2007 **| Time window for ascertaining screening rate:** Bi-annual **| Data source:** Administrative claims	60% of patients had at least two glucose tests in 2007	Kilbourne, 2011 [22]
**Glucose and Lipids Screening**		
**N:** 6601 **| % of study sample with schizophrenia or bipolar disorder:** Not reported **| Percent antipsychotic users:** 100% **| Study population:** Inpatient and outpatient California Medicaid recipients **| Screening year(s):** 2004–2005 **| Time window for ascertaining screening rate:** Six months prior-6 months after second generation antipsychotic initiation**| Data source:** Administrative claims	23% of patients had both a glucose test and a lipid test in the six months prior to antipsychotic initiation during 2004-2005	Barnett, 2010 [32]
	27% of patients had both a glucose test and a lipid test in the six months after antipsychotic initiation during 2004-2005	
**Panel 3: Screening for Dyslipidemia**		
**Any Lipid Screening**		
**N:** 200 **| % of study sample with schizophrenia or bipolar disorder:** 75% **| Percent antipsychotic users:** Not reported **| Study population:** Outpatients from psychiatric care centers in Baltimore, MD **| Screening year(s):** 2000 **| Time window for ascertaining screening rate:** Annual**| Data source**: Interview data	In 2000, 51% of patients reported having had their cholesterol checked in the past year	Dickerson 2003 [38]
**N:** 55436 **| % of study sample with schizophrenia or bipolar disorder:** 100% **| Percent antipsychotic users:** 100% **| Study population:** Inpatient and Outpatient Medicaid beneficiaries from California, Oregon, Tennessee and Utah **| Screening year(s):** 1998–2003 **| Time window for ascertaining screening rate:** 14 days prior-28 days after second generation antipsychotic initiation **| Data source:** Administrative claims	6% of those without schizophrenia and 9% of those with schizophrenia had a lipid test during 1998-2003	Morrato, 2008 [25]
	Compared to those without schizophrenia, those with schizophrenia had 1.4 times greater odds of having a lipid test during 1998–2003 (p < .05)	
**N:** 94 **| % of study sample with schizophrenia or bipolar disorder:** 100% **Percent antipsychotic users:** Not reported **| Study population:** Outpatient homeless shelter clinic users **| Screening year(s):** 1999–2000 **| Time window for ascertaining screening rate:** Annual **| Data source:** Medical chart review	55% of those with schizophrenia had a lipid test during 1999-2000	Folsom, 2002 [35]
**N:** 408 **| % of study sample with schizophrenia or bipolar disorder:** 100% **| Percent antipsychotic users:** 100% **| Study population:** Inpatient and outpatient Veterans **| Screening year(s):** 1999–2003 **| Time window for ascertaining screening rate:** Four years **| Data source:** Administrative claims	85% of patients had at least one lipid test during 1999-2003	Weissman, 2006 [31]
	27% of patients had only one lipid test during 1999-2003	
**N:** 64,490 **| % of study sample with schizophrenia or bipolar disorder:** 51% mood disorders, including bipolar disorder**| Percent antipsychotic users:** Not reported **| Study population:** Outpatient Veterans from New England Health Care System **| Screening year(s):** 2000–2001 **| Time window for ascertaining screening rate:** Annual **| Data source:** Administrative claims	85% of patients with mental illness had a lipid test, compared to 78% of patients without mental illness in 2001	Kaplowitz, 2006 [30]
	Compared to those without SMI, those with SMI had 2.73 times greater odds of having a lipid test in 2001 (p < .05)	
**N:** 18876 **| % of study sample with schizophrenia or bipolar disorder:** 3.4% schizophrenia, 49.3% mood disorders, including bipolar disorder**| Percent antipsychotic users:** 100% **| Study population:** Inpatient and outpatient commercial health insurance beneficiaries **| Screening year(s):** 2001–2006 **| Time window for ascertaining screening rate:** 30 days prior-30 days after second generation antipsychotic initiation **| Data source:** Administrative claims	The mean lipid testing rate for antipsychotic-treated patients was 8% during 2001-2006	Morrato, 2009 [27]
**N:** 2204**| % of study sample with schizophrenia or bipolar disorder:** 100% **| Percent antipsychotic users:** 100% **| Study population:** Inpatient and outpatient Kansas Medicaid beneficiaries **| Screening year(s):** 2003**| Time window for ascertaining screening rate:** Annual **| Data source:** Administrative claims	10% of patients had a lipid test during 2002-2003	Moeller, 2011 [26]
**N:** 1646**| Diagnoses:** Schizophrenia **| Percent antipsychotic users:** 100% **| Study population:** Inpatient and outpatient Kansas Medicaid beneficiaries **| Screening year(s):** 2005–2007 **| Time window for ascertaining screening rate:** Annual **| Data source:** Administrative claims	53% of patients had a lipid test during 2005-2007	
**N:** 6601 **| % of study sample with schizophrenia or bipolar disorder:** Not reported**| Percent antipsychotic users:** 100% **| Study population:** Inpatient and outpatient California Medicaid recipients **| Screening year(s):** 2004–2005 **| Time window for ascertaining screening rate:** Six months prior-6 months after second generation antipsychotic initiation **| Data source:** Administrative claims	39% of patients had a lipid test in the six months prior to antipsychotic initiation during 2004-2005	Barnett, 2010 [32]
	43% of patients had a lipid test in the six months after antipsychotic initiation during 2004-2005	
**N:** 46,430 **| % of study sample with schizophrenia or bipolar disorder:** 100% **| Percent antipsychotic users:** Not reported **| Study population:** Inpatient or outpatient veterans with hyperlipidemia participating in National Psychosis Registry **| Screening year(s):** 2005 **| Time window for ascertaining screening rate:** Annual **| Data source:** Medical chart review	90% of those with SMI, 94% of those with depression, and 94% of those without a psychiatric disorder had a lipid test in 2005 (p < .001)	Kilbourne, 2008 [34]
**N:** 40600 **| % of study sample with schizophrenia or bipolar disorder:** 100% **|Percent antipsychotic users:** 100% **| Study population:** Outpatient Veterans participating in the National Psychosis Registry**| Screening year(s):** 2007 **| Time window for ascertaining screening rate:** Annual **| Data source:** Administrative claims	37% of patients had a lipid test in 2007	Kilbourne, 2011 [22]
**Low Density Lipoprotein (LDL) Screening**		
**N:** 408 **| % of study sample with schizophrenia or bipolar disorder:** 100% **| Percent antipsychotic users:** 100% **| Study population:** Inpatient and outpatient Veterans **| Screening year(s):** 1999–2003 **| Time window for ascertaining screening rate:** four years**| Data source:** Administrative claims	74% of patients had at least one LDL test during 1999-2003	Weissman, 2006 [31]
	28% of patients had only one LDL test during 1999-2003	
**Triglyceride Screening**		
**N:** 408 **| % of study sample with schizophrenia or bipolar disorder:** 100% **| Percent antipsychotic users:** 100% **| Study population:** Inpatient and outpatient Veterans **| Screening year(s):** 1999–2003 **| Time window for ascertaining screening rate:** four years **| Data source:** Administrative claims	85% of patients had at least one triglycerides test during 1999-2003	Weissman, 2006 [31]
	27% of patients had only one triglycerides test during 1999-2003	
**Panel 4: Screening for Hypertension**		
**Any Blood Pressure Assessment**		
**N:** 200 **| % of study sample with schizophrenia or bipolar disorder:** 75%**| Percent antipsychotic users:** Not reported **| Study population:** Outpatients from psychiatric care centers in Baltimore, MD **| Screening year(s):** 2000 **| Time window for ascertaining screening rate:** Annual**| Data source**: Interview data	In 2000, 88% of patients reported having had their blood pressure checked in the past year	Dickerson 2003 [38]
**N:** 40600 **| % of study sample with schizophrenia or bipolar disorder:** 100% **|Percent antipsychotic users:** 100% **| Study population:** Outpatient Veterans participating in the National Psychosis Registry **| Screening year(s):** 2007 **| Time window for ascertaining screening rate:** Annual **| Data source:** Administrative claims	79% of patients had blood pressure assessed in 2007	Kilbourne, 2011 [22]

**Table 3:** **Adherence to national quality guidelines in persons with serious mental illness and the overall population**

USPSTF recommendation [ 13 ]	Studies (N)	Measures abstracted(N)	Population with SMI	Overall population
			Range in screening rates	National estimates of percent screened
**Panel 1: Screening for Overweight and Obesity**				
Screening all adults for obesity	1	1	Screening rate: 76% [22] Screening year: 2007	National estimates: 50% [23] - 71% [24] (2010)
**Panel 2: Screening for Diabetes**				
Screening for type 2 diabetes in asymptomatic adults with sustained blood pressure greater than 135/80 mmHg	5	10	Lowest screening rate: 23% [25] Screening year(s): 1998-2003	National estimates: 94% [29] (2005–2007)
			Highest screening rate: 75% [26] Screening year(s): 2005-2007	
**Panel 3: Screening for Dyslipidemia**				
Screening for lipid disorders (Men: aged 35+; or 20-35+ if at increased risk for coronary heart disease; Women: aged 20+ if at increased risk for coronary heart disease)	10	17	Lowest screening rate: 6% [25] Screening year(s): 1998-2003	National estimates: 68% [36] (1999–2006) - 79% [24] (2011)
			Highest screening rate: 90% [34] Screening year(s): 2005	
**Panel 4: Screening for Hypertension**				
Screening for high blood pressure in adults	2	2	Lowest screening rate: 79% [54] Screening year(s): 2007	National estimates: 59% [24] - 64% [40] (2010)
			Highest screening rate: 88% [38] Screening year(s): 2000	

### Diabetes mellitus (Panel 2)

The USPSTF recommends screening for diabetes mellitus in asymptomatic adults with sustained blood pressure (either treated or untreated) greater than 135/80 mmHg [13]. For antipsychotic users with SMI, the ADA/APA consensus panel recommends screening for diabetes mellitus at time of antipsychotic initiation, three months after initiation of a new antipsychotic medication, and annually thereafter [14].

In our review, we found six studies that measured diabetes mellitus screening in the population with SMI, totaling 12 measures. One hundred percent of the study population was taking antipsychotics for nine of the measures, 22% of the population was taking antipsychotics for one measure, and 0% of the population was taking antipsychotics for two measures. Glucose screening ranged from 18%-75%, and glucose and lipids screening ranged from 23%-27%. Glucose screening rates tended to increase over time, with 18%-29% of patients receiving screening between 1998–2003, [25,26] and 60%-75% receiving screening from 2006–2007 [22,26]. Low screening rates occurred in both commercial health insurance and Medicaid beneficiaries; testing rates below 30% among antipsychotic users were found in populations of commercial health insurance beneficiaries from 2001-2006 [27] and Medicaid beneficiaries with SMI from 2000–2003 [25,26]. In contrast, another study found that 60% of veterans with SMI using antipsychotics had at least two glucose tests in 2007 (Table 2) [28]. The time windows for ascertaining diabetes mellitus screening rates ranged from rates calculated in the 14 days prior to and 28 days after initiation of second-generation antipsychotics to annual rates. Screening rates tended to be lower when shorter time windows were employed. In comparison to the study population with SMI, from 2005–2007, 94% of patients from a Midwest academic physician group who met USPSTF guidelines for diabetes screening received a glucose test (Table 3) [29].

### Dyslipidemia (Panel 3)

The USPSTF recommends lipid screening for men aged 35 and older, men aged 20–35 if they are at increased risk of coronary heart disease, and women aged 20 and older if they are at increased risk of coronary heart disease [13]. For antipsychotic users with SMI, the ADA/APA consensus panel recommends screening for dyslipidemia at time of antipsychotic initiation, three months after antipsychotic initiation, and every five years thereafter [14].

In our review, we found 10 studies that measured dyslipidemia screening in the population with SMI, totaling 12 measures. One hundred percent of the population was taking antipsychotics for 13 of the measures, 22% of the population were taking antipsychotics for one of the measures, and antipsychotic use was not reported for three of the measures. Estimated rates of lipid testing among antipsychotic users with SMI ranged from 8% of beneficiaries in four commercial health plans during 2001-2006 [27] to 85% of veterans during 2000–2001 [30]. In the majority of studies, lipid screening rates among antipsychotic users were less than 50% (Table 2) [25-28,31-35]. One study measured low density lipoprotein (LDL) screening and triglyceride screening, finding that 74% of veterans had at least one LDL test and 85% had at least one triglyceride test during 1999–2003 [31]. The time windows for ascertaining dyslipidemia screening rates varied, ranging from the period spanning 14 days prior-28 days after initiation of second-generation antipsychotic medication to four years. In comparison to the population with SMI, 68%-79% of visits to office-based physicians included testing for lipids or cholesterol among high-risk groups (Table 3) [36,37].

### Hypertension (Panel 4)

The USPSTF recommends screening for hypertension in adults aged 18 and older. For antipsychotic users with SMI, [13] the ADA/APA consensus panel recommends screening for hypertension at time of antipsychotic initiation, three months after antipsychotic initiation, and annually thereafter [14].

In our review, we found two studies that measured hypertension screening in the population with SMI, totaling two measures. One hundred percent of the population was taking antipsychotics for one measure, and antipsychotic use was not reported for the other measure. Screening and monitoring rates for hypertension were generally high. Both measures showed rates of 79% or higher for any blood pressure measurement (Table 2) [22,38]. The time windows used to ascertain hypertension screening rates were one year [22] and four years, [38] respectively. In comparison to the study populations with SMI, in 2010, 59%-64% of US adults’ visits to the doctor included a blood pressure check (Table 3) [39,40].

In summary, we found that rates of cardiovascular screening varied considerably in the studies reviewed. Only one study measured overweight and obesity, and it found that 76% of patients with schizophrenia has BMI assessed in a given year. Rates of screening for diabetes mellitus ranged from 18% [25] to 75% [26]. Screening for dyslipidemia among adults with SMI ranged from 8% [25] to 85%, [31] Finally, screening for hypertension ranged from 79% [22] to 88% [38]. Estimates of screening rates for obesity and hypertension in the population with SMI were comparable – or higher, in the case of hypertension – to estimates of national screening rates for these conditions. For diabetes and dyslipidemia, the highest screening rates abstracted from studies of the SMI population were comparable to national rates, but the lowest rates were significantly lower. For example, national estimates of dyslipidemia screening range from 68% to 79%, and the lowest dyslipidemia screening rate in the population with SMI abstracted from the studies we reviewed was 6%. While we provide these general population rates for comparison, it is important to understand differences in time period, patient mix and settings across these studies in interpreting screening in SMI and non-SMI populations. Moreover, expert guidelines for persons with SMI taking antipsychotics recommend more frequent screening for cardiovascular risk factors than guidelines for the overall US population [14].

## Discussion

We found that rates of screening for cardiovascular risk factors in the population with SMI varied considerably in the studies reviewed. Much of this variation is likely attributable to health system differences, such as whether or not a given system has a standard protocol for screening for cardiovascular risk factors among antipsychotic users. Implementation of standard screening protocols is feasible and an effective tool for improving rates of screening. For example, six recent studies showed significant improvements in rates of screening for obesity in patients with SMI following implementation of standard screening protocols [41-46].

While health system differences may account for some of the variation in screening rates across the studies we reviewed, other important factors may help to explain this variability. Screening rates may change over time due to secular trends in medical practice. Variation in screening rates may also be due to differences in measurement technique (e.g. administrative claims data versus chart review) and the time window used to ascertain the screening rate. To be included in our review, study populations had to include – but were not limited to – persons with schizophrenia and bipolar disorder. Differences in diagnostic distribution within study populations could also influence variation in screening rates; however, the majority of study populations reviewed were comprised primarily of persons with schizophrenia and bipolar disorder using antipsychotic medications and no discernable patterns in screening rates by diagnostic composition of study sample were observed in our results.

Consistent with prior work, the results of our review suggest that rates measured over short time windows, for example in the 30 days following initiation of antipsychotic medication, tend to be lower than rates measured over longer periods [47]. In addition, rates are likely influenced by the frequency of patients’ encounters with the healthcare system. Patients who are frequent users of medical care services are more likely to be screened [48]. In addition, prior work has shown that individuals with more pre-existing risk factors for a condition are more likely to be screened for that condition [48]. Screening rates may be higher among those with pre-existing risk factors because providers prioritize screening among patients at heightened risk. This group may also be sicker and therefore more likely to use health services – increasing their chances of being screened due to increased contact with the healthcare system [48].

Variation in rates of screening for cardiovascular risk factors in the population with SMI is likely caused by multiple factors, including providers’ experience treating persons with SMI, [49] co-located versus geographically separate medical and mental health services, [18] and degree of continuity and coordination among medical care providers [50,51]. Consistent with studies evaluating quality of care conducted in the overall US population, [52,53] screening rates for cardiovascular risk factors among those with SMI tended to be high in the Veterans Health Administration (VHA) [22,30,31,54-56]. This is likely due at least partly to the VHA’s ability, as a national system, to use electronic patient data to trigger standard screening protocols for cardiovascular risk factors [57]. Ongoing initiatives to implement electronic health records in other health systems may provide an important tool to improve quality of medical care for persons with SMI [58].

The ongoing implementation of the Affordable Care Act presents a multitude of opportunities to improve screening for and treatment of cardiovascular risk factors among persons with SMI through initiatives such as Accountable Care Organizations (ACOs), Medicaid Health Homes, and state demonstration projects to integrate care for persons with SMI who are dually eligible for Medicare and Medicaid [50]. All three initiatives focus on coordinating care for populations with complex health needs, such as adults with SMI, to ensure that those with positive screenings receive appropriate follow-up treatment. Additionally, these initiatives rely heavily on health information technology, which can facilitate clinical decision support interventions and improve care coordination and patient outcomes [59-61]. Rigorous evaluation of these efforts is critical in order to understand how these new initiatives can best address the high burden of medical conditions and improve long-term health outcomes in the population with SMI.

Health homes, Accountable Care Organizations, and other integrated care and quality improvement initiatives use performance metrics to measure health system performance and, increasingly, determine rates of reimbursement and shared savings for physicians [62]. Quality measurement can be an important tool to drive quality improvement in the healthcare system. In response to the increased risk of metabolic syndrome associated with use of antipsychotics, the National Committee for Quality Assurance (NCQA), a private organization dedicated to improving the quality of health care in the US, released new quality measures in 2014 relating to diabetes and cardiovascular disease screening and monitoring for adults with SMI who are using antipsychotic medications [63]. More specifically, the NCQA defines three new measures: 1) diabetes screening for people with schizophrenia or bipolar disorder who are using antipsychotic medications; 2) diabetes monitoring for people with diabetes and schizophrenia; and 3) cardiovascular monitoring for people with cardiovascular disease and schizophrenia. Measurement of diabetes and cardiovascular disease screening and monitoring for adults with SMI will help to elucidate opportunities for improvement.

To our knowledge, this is the first study to provide a comprehensive review and compilation of screening rates for cardiovascular risk factors in the US population with SMI. The results of our review have several implications. First, the variation in screening rates suggests that researchers and practitioners interested in improving screening for cardiovascular conditions in this vulnerable population need to take into account unique healthcare setting, measurement, and population characteristics when designing and evaluating screening programs. Second, our results suggest that rates of screening for cardiovascular risk factors are very low in some study populations of persons with SMI, suggesting a need for improved implementation of screening in some settings. Third and perhaps most critically, our review identified considerable variation in the measurement and reporting of screening for cardiovascular risk factors in the population with SMI, making it difficult to compare screening rates across studies and with national rates. Given growing national interest in improving care and reducing costs for the population with SMI, there may be opportunities moving forward to standardize measurement and reporting of cardiovascular risk factor screening rates in this population. For example, 95% of persons with schizophrenia are insured by government programs: Medicaid and the Veteran’s Health Administration [64]. These insurers are already required to report multiple administrative-claims based measures of care quality to the federal government. Requiring these programs to report standardized measures of cardiovascular risk screening – for example the new NCQA measures described above – for beneficiaries with SMI would provide a critical tool for quality improvement and allow researchers and policy makers to monitor national screening rates over time.

These results should be interpreted in the context of several limitations. First, the screening measures abstracted from studies were not always directly comparable with one another or with national measures. The timing of measures varied, with some studies measuring a screening indicator within a one year period and others measuring over a longer period of time. Additionally, the patient characteristics of the included studies varied, which could have implications for receipt of cardiovascular screening tests as patient demographic characteristics have associated with receipt of preventive services in prior research [65], it is possible that our search strategy missed relevant articles, although to minimize this risk we searched the reference list of included articles. In addition, because our study does not include interventions or randomized trials, we did not systematically assess the internal validity of the included studies, as is standard in systematic reviews of clinical trials. Screening rates extracted from the studies in our review may be subject to measurement error, particularly those that estimate screening using administrative claims data. Prior research has shown that claims data underestimates services for some chronic conditions, such as obesity. To aid in interpretation of results, we presented information about data source, measurement specification, and the time window in which the screening rate was measured. Other potential sources of bias to our results include selective reporting within studies and publication bias, but we were unable to identify the extent of these threats to validity based on the content of the studies reviewed.

## Conclusion

In this comprehensive literature review, rates of screening for cardiovascular risk factors in the population with SMI varied considerably by time period and study population. There is substantial need for improvement in providing routine screening and appropriate follow-up care for cardiovascular risk factors among some subpopulations with SMI. Research suggests that implementation of standard screening protocols triggered by SMI diagnosis or antipsychotic use are promising avenues for early identification of cardiovascular risk factors in the population with SMI [66]. Future research should consider how best to connect screening to the other processes of care – including diagnosis, treatment, and monitoring – necessary in order to improve health outcomes for people with SMI.

## Additional files

Additional file 1:
**Abstraction Tool.**

Additional file 2:
**Search strategies.**

## Abbreviations

SMI: Serious mental illness
US: United States
USPSTF: United States preventive services task force
ADA/APA: Consensus panel: American diabetes association, American psychiatric association, American Association of clinical edocrinologists, and North American association for the study of obesity
BMI: Body mass index
LDL: Low-density lipoprotein
VHA: Veteran’s health administration
ACOs: Accountable care organizations

## References

[1] Brown S (1997). Excess mortality of schizophrenia. A meta-analysis. Br J Psychiatry.

[2] Saha S, Chant D, McGrath J (2007). A systematic review or mortality in schizophrenia: is the differential mortality gap worsening over time?. Arch Gen Psychiatry.

[3] Daumit GL, Anthony CB, Ford DE, Fahey M, Skinner EA, Lehman AF, Hwang W, Steinwachs DM (2010). Pattern of mortality in a sample of maryland residents with severe mental illness. Psychiatry Res.

[4] Colton CP, Ronald W, Manderscheid P (2006). Congruencies in increased mortality rates, years of life lost, and causes of death among public mental health clients in eight states. Prev Chronic Dis.

[5] Osby U, Correia N, Brandt L, Ekbom A, Sparen P (2000). Mortality and causes of death in schizophrenia in Stockholm county, Sweden. Schizophr Res.

[6] Osborn DP, Levy G, Nazareth I, Petersen I, Islam A, King MB (2007). Relative risk of cardiovascular and cancer mortality in people with severe mental illness from the United Kingdom’s general practice database. Arch Gen Psychiatry.

[7] Newcomer JW, Hennekens CH (2007). Severe mental illness and risk of cardiovascular disease. JAMA.

[8] Allison D, Newcomer JW, Dunn AL (2009). Obesity among those with mental disorders: a national institute of mental health meeting report. Am J Prev Med.

[9] McDevitt J, Snyder M, Miller A, Wilbur J (2006). Perceptions of bariers and benefits to physical activity among outpatients in psychiatric rehabilitation. J Nurs Scholarsh.

[10] Casagrande SS, Anderson CA, Appel L, Jerome G, Dickerson FB, Daumit GL (2011). Dietary intake of adults with serious mental illness. Psychiatr Rehabil J..

[11] Compton MT, Daumit GL, Druss BG (2006). Cigarette smoking and overweight/obesity among individuals with serious mental illness: a preventive perspective. Harv Rev Psychiatry.

[12] Allison DB, Mentore JL, Heo M (1999). Antipsychotic-induced weight gain: a comprehensive research synthesis. Am J Psychiatry.

[13] United States Preventive Services Task Force (USPSTF): Reommendations for Adults. http://www.uspreventiveservicestaskforceorg/adultrechtm 2013.

[14] American Diabetes Association (2004). American psychiatric association, American association of clinical edocrinologists, North American association for the study of obesity: consensus development conference for antipsychotic drugs and obesity and diabetes. Diabetes Care.

[15] Kreyenbuhl J, Dickerson FB, Medoff DR, Brown CH, Goldberg RW, Fang L (2006). Extent and management of cardiovascular risk factors in patients with type 2 diabetes and serious mental illness. J Nerv Ment Dis.

[16] Sokal J, Messias E, Dickerson FB, Kreyenbuhl J, Brown CH, Goldberg RW (2004). Comorbidity of medical illnesses among adults with serious mental illness who are receiving community psychiatric services. J Nerv Ment Dis.

[17] American Diabetes Association (2004). Screening for Type 2 Diabetes. Diabetes Care.

[18] Butler M, Kane RL, McAlpine D, al; e: Integration of Mental Health/Substance Abuse and Primary Care. Prepared by the Minnesota Evidence-Based Practice Center under contract 290-02-0009. AHRQ pub no 09-E003. Rockville, MD, Agency for Healthcare Research and Quality, October 2008. Available at www.ahrq.gov/clinic/tp/mhsapctp.htm.

[19] Ganguli R (2011). Comorbidities of obesity in serious mental illness. Psychiatr Ann.

[20] Miller BJ, Paschall CB, Svendsen DP (2006). Mortality and medical comorbidity among patients with serious mental illness. Psychiatr Serv.

[21] Baillargeon JG, Paar DP, Wu H, Giordano TP, Murray O, Raimer BG (2008). Psychiatric disorders, HIV infection and HIV/hepatitis co-infection in the correctional setting. AIDS Care - Psychological and Socio-Medical Aspects of AIDS/HIV.

[22] Kilbourne AM, Lai Z, Bowersox N, Pirraglia P, Bauer MS (2011). Does colocated care improve access to cardiometabolic screening for patients with serious mental illness?. Gen Hosp Psychiatry.

[23] Ma J, Xaio L, Stafford RS (2009). Adult obesity and office-based quality of car ein the US. Obesity.

[24] National Ambulatory Medical Care Survey: National Ambulatory Medical Care Survey: 2010 Summary Tables. http://www.cdcgov/nchs/data/ahcd/namcs_summary/2010_namcs_web_tablespdf 2010.

[25] Morrato EH, Newcomer JW, Allen RR, Valuck RJ (2008). Prevalence of baseline serum glucose and lipid testing in users of second-generation antipsychotic drugs: a retrospective, population-based study of medicaid claims data. J Clin Psychiatry.

[26] Moeller KE, Rigler SK, Mayorga A, Nazir N, Shireman TI (2011). Quality of monitoring for metabolic effects associated with second generation antipsychotics in patients with schizophrenia on public insurance. Schizophr Res.

[27] Morrato EH, Newcomer JW, Kamat S, Baser O, Harnett J, Cuffel B (2009). Metabolic screening after the American diabetes association’s consensus statement on antipsychotic drugs and diabetes. Diabetes Care.

[28] Kilbourne AM, Greenwald DE, Hermann RC, Charns MP, McCarthy JF, Yano EM (2010). Financial incentives and accountability for integrated medical care in department of veterans affairs mental health programs. Psychiatric services (Washington, DC).

[29] Sheehy AM, Flood GE, Tuan WJ, Liou JI, Coursin DB, Smith MA (2010). Analysis of guidelines for screening diabetes mellitus in an ambulatory population. Mayo Clin Proc.

[30] Kaplowitz RA, Scranton RE, Gagnon DR, Cantillon C, Cantillon C, Levenson JW (2006). Health care utilization and receipt of cholesterol testing by veterans with and those without mental illness. Gen Hosp Psychiatry.

[31] Weissman EM, Zhu CW, Schooler NR, Goetz RR, Essock SM (2006). Lipid monitoring in patients with schizophrenia prescribed second-generation antipsychotics. J Clin Psychiatry.

[32] Barnett M, VonMuenster S, Wehring H, Popish S, McDonald K, Walker VM (2010). Assessment of monitoring for glucose and lipid dysregulation in adult Medi-Cal patients newly started on antipsychotics. Ann Clin Psychiatry.

[33] Pirraglia PA, Rowland E, Wu WC, Friedmann PD, O’Toole TP, Cohen LB (2012). Benefits of a primary care clinic co-located and integrated in a mental health setting for veterans with serious mental illness. Prev Chronic Dis.

[34] Kilbourne AM, Welsh D, McCarthy JF, Post EP, Blow FC (2008). Quality of care for cardiovascular disease-related conditions in patients with and without mental disorders. J Gen Intern Med.

[35] Folsom DP, McCahill M, Bartels S, Lindamer LA, Ganiats TG, Jeste DV (2002). Medical comorbidity and receipt of medical care by older homeless people with schizophrenia or depression. Psychiatr Serv.

[36] Kuklina EV, Yoon PW, Keenan NL (2010). Prevalence of coronary heart disease risk factors and screening for high cholesterol levels among young adults, United States, 1999–2006. Ann Fam Med.

[37] Behavioral Risk Factor Surveillance System: Cholesterol Awareness-2011 [http://apps.nccd.cdc.gov/brfss/list.asp?cat=CA&yr=2011&qkey=8061&state=All].

[38] Dickerson FB, McNary W, Brown CH, Kreyenbuhl J, Goldberg RW, Dixon LB (2003). Somatic healthcare utilization among adults with serious mental illness who are receiving community psychiatric services. Med Care.

[39] National Ambulatory Medical Care Survey: 2010 Summary Tables [http://www.cdc.gov/nchs/data/ahcd/namcs_summary/2010_namcs_web_tables.pdf].

[40] National Hospital Ambulatory Medical Care Survey: 2010 Outpatient Department Summary Tables [http://www.cdc.gov/nchs/data/ahcd/nhamcs_outpatient/2010_opd_web_tables.pdf].

[41] O’Callaghan C, Liew AYL, Yusof MSD, Duffy R, Breen EG, Kinsley B (2011). Screening for metabolic syndrome in long-term psychiatric illness: audit of patients receiving depot antipsychotic medication at a psychiatry clinic. The Eur J Psychol Psychiatry.

[42] Barnes TR, Paton C, Hancock E, Cavanagh MR, Taylor D, Lelliott P (2008). Screening for the metabolic syndrome in community psychiatric patients prescribed antipsychotics: a quality improvement programme. Acta Psychiatrica Scandinavica.

[43] Bobes J, Alegria AA, Saiz-Gonzalez MD, Barber I, Perez JL, Saiz-Ruiz J (2011). Change in psychiatrists’ attitudes towards the physical health care of patients with schizophrenia coinciding with the dissemination of the consensus on physical health in patients with schizophrenia. Eur Psychiatry.

[44] Harrison MR, McMillan CF, Dickinson T (2012). Service innovation: a comparison of two approaches for physical screening of psychiatric inpatients. Int J Psychiatry Clin Pract.

[45] Wiechers IR, Viron M, Stoklosa J, Freudenreich O, Henderson DC, Weiss A (2012). Impact of a metabolic screening bundle on rates of screening for metabolic syndrome in a psychiatry resident outpatient clinic. Acad Psychiatry.

[46] Osborn DP, Nazareth I, Wright CA, King MB (2010). Impact of a nurse-led intervention to improve screening for cardiovascular risk factors in people with severe mental illnessesPhase-two cluster randomised feasibility trial of community mental health teams. BMC Health Serv Res.

[47] Morrato EH, Druss B, Hartung DM, Valuck RJ, Allen R, Campagna E (2010). Metabolic testing rates in 3 state Medicaid programs after FDA warnings and ADA/APA recommendations for second-generation antipsychotic drugs. Arch Gen Psychiatry.

[48] Morrato EH, Druss BG, Hartung DM, Valuck RJ, Thomas D, Allen R (2011). Small area variation and geographic and patient-specific determinants of metabolic testing in antipsychotic users. Pharmacoepidemiol Drug Saf.

[49] Hodges B, Inch C, Silver I (2001). Improving the psychiatric knowledge, skills, and attitudes of primary care physicians, 1950–2000: a review. Am J Psychiatr.

[50] Druss BG, Mauer BJ (2010). Health care reform and care at the behavioral health-primary care interface. Psychiatr Serv.

[51] Michell AJ, Malone D, Doebbeling CC (2009). Quality of medical care for people with and without comorbid mental illness and substance misuse: systematic review of comparative studies. Br J Psychiatry.

[52] Asch SM, McGlynn EA, Hogan MM, Hayward RA, Shekelle P, Rubenstein L (2004). Comparison of quality of care for patients in the veterans health administration and patients in a national sample. Ann Intern Med.

[53] Jha AK, Perlin JB, Kizer KW, Dudley RA (2003). Effect of the transformation of the veterans affairs health care system on the quality of care. N Engl J Med.

[54] Kilbourne AM, Pirraglia PA, Lai Z, Bauer MS, Charns MP, Greenwald D (2011). Quality of general medical care among patients with serious mental illness: does colocation of services matter?. Psychiatric services (Washington, DC).

[55] Goodrich DE, Lai ZS, Lasky E, Burghardt AR, Kilbourne AM (2010). Access to weight loss counseling services among patients with bipolar disorder. J Affect Disord.

[56] Matthews AM, Huckans MS, Blackwell AD, Hauser P (2008). Hepatitis C testing and infection rates in bipolar patients with and without comorbid substance use disorders. Bipolar Disord.

[57] Kizer KW, Dudley RA (2009). Extreme makeover: transformation of the veterans health care system. Annu Rev Public Health.

[58] Hillestad R, Bigelow J, Bower A, Girosi F, Meili R, Scoville R (2005). Can electronic medical record systems transform health care? potential health benefits, savings. And Costs Health Affairs.

[59] Romano MJ, Stafford RS (2011). Electronic health records and clinical decision support systems: impact on national ambulatory care quality. Arch Intern Med.

[60] Reed M, Huang J, Brand R, Graetz I, Neugebauer R, Fireman B (2013). Implementation of an outpatient electronic health record and emergency department visits, hospitalizations, and office visits among patients with diabetes. JAMA.

[61] Jaspers MW, Smeulers M, Vermeulen H, Peute LW (2011). Effects of clinical decision-support systems on practitioner performance and patient outcomes: a synthesis of high-quality systematic review findings. J Am Med Inform Assoc.

[62] Bao Y, Casalino LP, Pincus HA (2013). Behavioral health and health care reform models: patient centered medical home, health home, and accountable care organization. J Behav Heal Serv Res.

[63] (NCQA). NCfQA: HEDIS 2014 (2013). Healthcare Effectiveness Data and Information Set. In., vol. 1.

[64] Khaykin E, Eaton WW, Ford DE, Anthony CB, Daumit GL (2010). Health insurance coverage among persons with schizophrenia in the United States. Psychiatric services (Washington, DC).

[65] Fiscella K, Franks P, Gold MR, Clancy CM (2000). Inequality in quality: addressing socioeconomic, racial, and ethnic disparities in health care. JAMA.

[66] Mitchell A, Delaffon V, Vancampfort D, Correll C, De Hert M (2012). Guideline concordant monitoring of metabolic risk in people treated with antipsychotic medication: systematic review and meta-analysis of screening practices. Psychol Med.

