# Supplementary material for: Screening for cardiovascular risk factors in adults with serious mental illness: a review of the evidence
Source: BMC Psychiatry. 2015 Mar 21;15:55. doi: 10.1186/s12888-015-0416-y (PMC4376086; doi:10.1186/s12888-015-0416-y)
Supplement: Additional file 1: — Abstraction Tool. [file 12888_2015_416_MOESM1_ESM.docx]

**Appendix A: Search Strategies**

**PUBMED**

((“access to health care”[tiab] OR “accessibility of health services”[tiab] OR “availability of health services”[tiab] OR “health services availability”[tiab] “screening rate” [tiab] OR “screening rates” [tiab] OR “rates of screening” [tiab] OR “rate of service use” [tiab] OR “rates of service use” [tiab] OR “access” [tiab] OR “access to care” [tiab] OR “access to primary care” [tiab])

AND

(Schizophrenia[mh] OR “Paranoid Disorders”[mh] OR “Psychotic Disorders”[mh] OR “Bipolar Disorder”[mh:noexp] OR “Depressive Disorder, Major”[mh] OR “Serious Mental Illness”[tiab] OR “Serious Mental Illnesses”[tiab] OR “Severe Mental Illness”[tiab] OR “Severe Mental Illnesses”[tiab] OR “Seriously Mentally Ill”[tiab] OR “Severely Mentally Ill”[tiab] OR “Serious Mental Disorder”[tiab] OR “Serious Mental Disorders”[tiab] OR “Severe Mental Disorder”[tiab] OR “Severe Mental Disorders[tiab]” OR Schizophrenia[tiab] OR “Paranoid Disorders”[tiab] OR “Psychotic Disorders”[tiab] OR “Bipolar Disorder”[tiab] OR “Depressive Disorder, Major”[tiab] OR Schizophrenias[tiab] OR “Schizophrenic Disorders”[tiab] OR “Schizophrenic Disorder”[tiab] OR “Dementia Praecox”[tiab] OR “Paranoid Disorder”[tiab] OR “Paranoid Psychoses”[tiab] OR “Psychotic Disorder”[tiab] OR “Psychosis”[tiab] OR “Psychoses”[tiab] OR “Schizoaffective Disorder”[tiab] OR “Schizoaffective Disorders”[tiab] OR “Schizophreniform Disorders”[tiab] OR “Schizophreniform Disorder”[tiab] OR “Brief Reactive Psychoses”[tiab] OR “Brief Reactive Psychosis”[tiab] OR “Bipolar Disorders”[tiab] OR “Manic-Depressive Psychosis”[tiab] OR “Manic Depressive Psychosis”[tiab] OR “Bipolar Affective Psychosis”[tiab] OR “Manic-Depressive Psychoses”[tiab] OR Mania[tiab] OR Manias[tiab] OR “Manic State”[tiab] OR “Manic States”[tiab] OR “Bipolar Depression”[tiab] OR “Manic Disorder”[tiab] OR “Manic Disorders”[tiab] OR “Major Depressive Disorders”[tiab] OR “Major Depressive Disorder”[tiab] OR “Involutional Psychoses”[tiab] OR “Involutional Psychosis”[tiab] OR “Involutional Depression”[tiab] OR “Involutional Melancholia”[tiab] OR “Major Mental Illness”[tiab] OR “Major Mental Illnesses”[tiab] OR “Major Psychiatric Disorder”[tiab] OR “Major Psychiatric Disorders”[tiab] OR “Serious Psychiatric Disorder”[tiab] OR “Serious Psychiatric Disorders”[tiab] OR “Severe Psychiatric Disorder”[tiab] OR “Severe Psychiatric Disorders”[tiab])

AND

(“Cardiovascular Diseases”[mh] OR Overweight[mh] OR Hypertension[mh] OR Hyperlipidemias[mh] OR Cholesterol[mh] OR “Diabetes Mellitus”[mh:noexp] OR “Diabetes Mellitus, Type 2”[mh] OR “Prediabetic State”[mh] OR “Blood Glucose”[mh] OR “Hemoglobin A, Glycosylated”[mh] OR Neoplasms[mh] OR “Motor Activity”[mh] OR Diet[mh] OR “Nutritional Status”[mh] OR “Pulmonary Disease, Chronic Obstructive”[mh] OR “Metabolic Syndrome X”[mh] OR “Weight Loss”[mh] OR “Body Mass Index”[mh] OR “Cardiovascular Diseases”[tiab] OR “Cardiovascular Disease”[tiab] OR “Stroke”[tiab] OR “Heart Disease”[tiab] OR “Cardiac Diseases”[tiab] OR “Vascular Disease”[tiab] OR “Overweight”[tiab] OR “Obese”[tiab] OR “Obesity”[tiab] OR “Hypertension”[tiab] OR “High Blood Pressure”[tiab] OR “High Blood Pressures”[tiab] OR hyperlipidemia[tiab] OR Hyperlipidemias[tiab] OR Hyperlipemia[tiab] OR Lipidemia[tiab] OR Lipidemias[tiab] OR Lipemia[tiab] OR Lipemias[tiab] OR “Cholesterol”[tiab] OR “Diabetes Mellitus”[tiab] OR “Diabetes Mellitus, Type 2”[tiab] OR “Prediabetic State”[tiab] OR “Ketosis-Resistant Diabetes Mellitus”[tiab] OR “Non-Insulin-Dependent Diabetes Mellitus”[tiab] OR “Type 2 Diabetes Mellitus”[tiab] OR “Slow-Onset Diabetes Mellitus”[tiab] OR “Stable Diabetes Mellitus”[tiab] OR “Maturity-Onset Diabetes Mellitus”[tiab] OR “Maturity Onset Diabetes Mellitus”[tiab] OR “MODY”[tiab] OR “NIDDM”[tiab] OR “Adult-Onset Diabetes Mellitus”[tiab] OR “Blood Glucose”[tiab] OR “Blood Sugar”[tiab] OR “Hemoglobin”[tiab] OR “Motor Activity”[tiab] OR “Motor Activities”[tiab] OR “Physical Activity”[tiab] OR “Physical Activities”[tiab] OR “Locomotor Activity”[tiab] OR “Locomotor Activities”[tiab] OR “Exercise”[tiab] OR “Exercises”[tiab] OR “Physical Exercise”[tiab] OR “Physical Exercises”[tiab] OR “Isometric Exercises”[tiab] OR “Aerobic Exercise”[tiab] OR “Aerobic Exercises”[tiab] OR Diet[tiab] OR Nutrition[tiab] OR “Metabolic Syndrome”[tiab] OR “Metabolic Cardiovascular Syndrome”[tiab] OR “Weight Loss”[tiab] OR “Weight Losses”[tiab] OR “Weight Reduction”[tiab] OR “Weight Reductions”[tiab] OR “Body Mass Index”[tiab] OR “BMI”[tiab])

AND

(“United States”[mh] OR [Alabama](http://www.ncbi.nlm.nih.gov/mesh/68000407)[mh] OR Alaska[mh] OR Arizona[mh] OR Arkansas[mh] OR California[mh] OR Colorado[mh] OR Connecticut[mh] OR Delaware[mh] OR Florida[mh] OR Georgia[mh] OR Hawaii[mh] OR Idaho[mh] OR Illinois[mh] OR Indiana[mh] OR Iowa[mh] OR Kansas[mh] OR Kentucky[mh] OR Louisiana[mh] OR Maine[mh] OR Maryland[mh] OR Massachusetts[mh] OR Michigan[mh] OR Minnesota[mh] OR Mississippi[mh] OR Missouri[mh] OR Montana[mh] OR Nebraska[mh] OR Nevada[mh] OR “New Hampshire”[mh] OR “New Jersey”[mh] OR “New Mexico”[mh] OR “New York”[mh] OR “North Carolina” [mh] OR “North Dakota”[mh] OR Ohio[mh] OR Oklahoma[mh] OR Oregon[mh] OR Pennsylvania[mh] OR “Rhode Island”[mh] OR “South Carolina”[mh] OR “South Dakota”[mh] OR Tennessee[mh] OR Texas[mh] OR Utah[mh] OR Vermont[mh] OR Virginia[mh] OR Washington[mh] OR “West Virginia”[mh] OR Wisconsin[mh] OR Wyoming[mh] OR Chicago[mh] OR “New York City”[mh] OR Baltimore[mh] OR Philadelphia[mh] OR Boston[mh] OR “Los Angeles”[mh] OR “San Francisco”[mh] OR “New Orleans”[mh] OR “United States” [tiab] OR “US”[tiab] OR “USA”[tiab] OR “United States of America”[tiab] OR [Alabama](http://www.ncbi.nlm.nih.gov/mesh/68000407)[tiab] OR Alaska[tiab] OR Arizona[tiab] OR Arkansas[tiab] OR California[tiab] OR Colorado[tiab] OR Connecticut[tiab] OR Delaware[tiab] OR Florida[tiab] OR Georgia[tiab] OR Hawaii[tiab] OR Idaho[tiab] OR Illinois[tiab] OR Indiana[tiab] OR Iowa[tiab] OR Kansas[tiab] OR Kentucky[tiab] OR Louisiana[tiab] OR Maine[tiab] OR Maryland[tiab] OR Massachusetts[tiab] OR Michigan[tiab] OR Minnesota[tiab] OR Mississippi[tiab] OR Missouri[tiab] OR Montana[tiab] OR Nebraska[tiab] OR Nevada[tiab] OR “New Hampshire”[tiab] OR “New Jersey”[tiab] OR “New Mexico”[tiab] OR “New York”[tiab] OR “North Carolina” [tiab] OR “North Dakota”[tiab] OR Ohio[tiab] OR Oklahoma[tiab] OR Oregon[tiab] OR Pennsylvania[tiab] OR “Rhode Island”[tiab] OR “South Carolina”[tiab] OR “South Dakota”[tiab] OR Tennessee[tiab] OR Texas[tiab] OR Utah[tiab] OR Vermont[tiab] OR Virginia[tiab] OR Washington[tiab] OR “West Virginia”[tiab] OR Wisconsin[tiab] OR Wyoming[tiab] OR Chicago[tiab] OR “New York City”[ tiab] OR Baltimore[tiab] OR Philadelphia[tiab] OR Boston[tiab] OR “Los Angeles”[ tiab] OR “San Francisco”[ tiab] OR “New Orleans”[tiab] OR “United States” OR “US” OR “USA” OR “United States of America” OR [Alabama](http://www.ncbi.nlm.nih.gov/mesh/68000407) OR Alaska OR Arizona OR Arkansas OR California OR Colorado OR Connecticut OR Delaware OR Florida OR Georgia OR Hawaii OR Idaho OR Illinois OR Indiana OR Iowa OR Kansas OR Kentucky OR Louisiana OR Maine OR Maryland OR Massachusetts OR Michigan OR Minnesota OR Mississippi OR Missouri OR Montana OR Nebraska OR Nevada OR “New Hampshire” OR “New Jersey”[ OR “New Mexico” OR “New York” OR “North Carolina” OR “North Dakota” OR Ohio OR Oklahoma OR Oregon OR Pennsylvania OR “Rhode Island” OR “South Carolina” OR “South Dakota” OR Tennessee OR Texas OR Utah OR Vermont OR Virginia OR Washington OR “West Virginia” OR Wisconsin OR Wyoming)

AND

("2000"[dp]: "3000"[dp])

NOT

(“randomized”[tiab] OR “trial”[tiab] OR “quality of life”[tiab] OR “effects”[tiab] OR “systematic review” OR “meta-analysis” OR “meta-analysis”))

**PSYCHINFO**

(“access to health care” OR TI “accessibility of health services” OR TI “availability of health services” OR TI “health services availability” OR TI “screening rate” OR TI “screening rates” OR TI “rates of screening” OR TI “rate of service use” OR TI “rates of service use” “access” OR TI “access to care” OR TI “access to primary care” OR AB “access to health care” OR AB “accessibility of health services” OR AB “availability of health services” OR AB “health services availability” OR AB “screening rate” OR AB “screening rates” OR AB “rates of screening” OR AB “rate of service use” OR AB “rates of service use” “access” OR AB “access to care” OR AB “access to primary care”)

AND

(DE “Schizophrenia” OR DE “Acute Schizophrenia” OR DE “Catatonic Schizophrenia” OR DE “Paranoid Schizophrenia” OR DE “Process Schizophrenia” OR DE “Schizophrenia (Disorganized Type)” OR DE “Schizophreniform Disorder” OR DE “Schizoaffective Disorder” OR DE “Undifferentiated Schizophrenia” OR DE “Psychosis” OR DE “Bipolar Disorder” OR DE “Major Depression” OR DE “Neuroleptic Drugs” OR TI “Serious Mental Illness” OR TI “Serious Mental Illnesses” OR TI “Severe Mental Illness” OR TI “Severe Mental Illnesses” OR TI “Seriously Mentally Ill” OR TI “Severely Mentally Ill” OR TI “Serious Mental Disorder” OR TI “Serious Mental Disorders” OR TI “Severe Mental Disorder” OR TI “Severe Mental Disorders” OR TI “Schizophrenia” OR TI “Paranoid Disorders” OR TI “Psychotic Disorders” OR TI “Bipolar Disorder” OR TI “Major Depressive Disorder” OR TI “Schizophrenias” OR TI “Schizophrenic Disorders” OR TI “Schizophrenic Disorder” OR TI “Dementia Praecox” OR TI “Paranoid Disorder” OR TI “Paranoid Psychoses” OR TI “Psychotic Disorder” OR TI “Psychosis” OR TI “Psychoses” OR TI “Schizoaffective Disorder” OR TI “Schizoaffective Disorders” OR TI “Schizophreniform Disorders” OR TI “Schizophreniform Disorder” OR TI “Brief Reactive Psychoses” OR TI “Brief Reactive Psychosis” OR TI “Bipolar Disorders” OR TI “Manic-Depressive Psychosis” OR TI “Manic Depressive Psychosis” OR TI “Bipolar Affective Psychosis” OR TI “Manic-Depressive Psychoses” OR TI “Mania” OR TI “Manias” OR TI “Manic State” OR TI “Manic States” OR TI “Bipolar Depression” OR TI “Manic Disorder” OR TI “Manic Disorders” OR TI “Major Depressive Disorders” OR TI “Major Depressive Disorder” OR TI “Involutional Psychoses” OR TI “Involutional Psychosis” OR TI “Involutional Depression” OR TI “Involutional Melancholia” OR TI “Antipsychotic” OR TI “Antipsyhotics” OR TI “Major Mental Illness” OR TI “Major Mental Illnesses” OR TI “Major Psychiatric Disorder” OR TI “Major Psychiatric Disorders” OR TI “Serious Psychiatric Disorder” OR TI “Serious Psychiatric Disorders” OR TI “Severe Psychiatric Disorder” OR TI “Severe Psychiatric Disorders”

OR AB “Serious Mental Illness” OR AB “Serious Mental Illnesses” OR AB “Severe Mental Illness” OR AB “Severe Mental Illnesses” OR AB “Seriously Mentally Ill” OR AB “Severely Mentally Ill” OR AB “Serious Mental Disorder” OR AB “Serious Mental Disorders” OR AB “Severe Mental Disorder” OR AB “Severe Mental Disorders” OR AB “Schizophrenia” OR AB “Paranoid Disorders” OR AB “Psychotic Disorders” OR AB “Bipolar Disorder” OR AB “Major Depressive Disorder” OR AB “Schizophrenias” OR AB “Schizophrenic Disorders” OR AB “Schizophrenic Disorder” OR AB “Dementia Praecox” OR AB “Paranoid Disorder” OR AB “Paranoid Psychoses” OR AB “Psychotic Disorder” OR AB “Psychosis” OR AB “Psychoses” OR AB “Schizoaffective Disorder” OR AB “Schizoaffective Disorders” OR AB “Schizophreniform Disorders” OR AB “Schizophreniform Disorder” OR AB “Brief Reactive Psychoses” OR AB “Brief Reactive Psychosis” OR AB “Bipolar Disorders” OR AB “Manic-Depressive Psychosis” OR AB “Manic Depressive Psychosis” OR AB “Bipolar Affective Psychosis” OR AB “Manic-Depressive Psychoses” OR AB “Mania” OR AB “Manias” OR AB “Manic State” OR AB “Manic States” OR AB “Bipolar Depression” OR AB “Manic Disorder” OR AB “Manic Disorders” OR AB “Major Depressive Disorders” OR AB “Major Depressive Disorder” OR AB “Involutional Psychoses” OR AB “Involutional Psychosis” OR AB “Involutional Depression” OR AB “Involutional Melancholia” OR AB “Antipsychotic” OR AB “Antipsyhotics” OR AB “Major Mental Illness” OR AB “Major Mental Illnesses” OR AB “Major Psychiatric Disorder” OR AB “Major Psychiatric Disorders” OR AB “Serious Psychiatric Disorder” OR AB “Serious Psychiatric Disorders” OR AB “Severe Psychiatric Disorder” OR AB “Severe Psychiatric Disorders”)

AND

(DE "Cardiovascular Disorders" OR DE "Cerebrovascular Disorders" OR DE "Overweight" OR DE "Obesity" OR DE "Body Mass Index" OR DE "Weight Loss" OR DE "Weight Gain" OR DE "Weight Control" OR DE "Hypertension" OR DE "Cholesterol" OR DE "Lipids" OR DE "Diabetes Mellitus" OR DE "Blood Sugar" OR DE "Hemoglobin" OR DE "Neoplasms" OR DE "Physical Activity" OR DE "Exercise" OR DE "Diets" OR DE "Eating Behavior" OR DE "Nutrition" OR DE "Metabolic Syndrome" OR TI “Cardiovascular Diseases” OR TI “Cardiovascular Disease” OR TI “Stroke” OR TI “Heart Disease” OR TI “Cardiac Diseases” OR TI “Vascular Disease” OR TI “Overweight” OR TI “Obese” OR TI “Obesity” OR TI “Hypertension” OR TI “High Blood Pressure” OR TI “High Blood Pressures” OR TI “Hyperlipidemia” OR TI “Hyperlipidemias” OR TI “Hyperlipemia” OR TI “Lipidemia” OR TI “Lipidemias” OR TI “Lipemia” OR TI “Lipemias” OR TI “Cholesterol” OR TI “Diabetes Mellitus” OR TI “Diabetes Mellitus, Type 2” OR TI “Prediabetic State” OR TI “Ketosis-Resistant Diabetes Mellitus” OR TI “Non-Insulin-Dependent Diabetes Mellitus” OR TI “Type 2 Diabetes Mellitus” OR TI “Slow-Onset Diabetes Mellitus” OR TI “Stable Diabetes Mellitus” OR TI “Maturity-Onset Diabetes Mellitus” OR TI “Maturity Onset Diabetes Mellitus” OR TI “MODY” OR TI “NIDDM” OR TI “Adult-Onset Diabetes Mellitus” OR TI “Blood Glucose” OR TI “Blood Sugar” OR TI “Hemoglobin” OR TI “Motor Activity” OR TI “Motor Activities” OR TI “Physical Activity” OR TI “Physical Activities” OR TI “Locomotor Activity” OR TI “Locomotor Activities” OR TI “Exercise” OR TI “Exercises” OR TI “Physical Exercise” OR TI “Physical Exercises” OR TI “Isometric Exercises” OR TI “Aerobic Exercise” OR TI “Aerobic Exercises” OR TI “Diet” OR TI “Nutrition” OR TI “Metabolic Syndrome” OR TI “Metabolic Cardiovascular Syndrome” OR TI “Weight Loss” OR TI “Weight Losses” OR TI “Weight Reduction” OR TI “Weight Reductions” OR TI “Body Mass Index” OR TI “BMI” OR AB “Cardiovascular Diseases” OR AB “Cardiovascular Disease” OR AB “Stroke” OR AB “Heart Disease” OR AB “Cardiac Diseases” OR AB “Vascular Disease” OR AB “Overweight” OR AB “Obese” OR AB “Obesity” OR AB “Hypertension” OR AB “High Blood Pressure” OR AB “High Blood Pressures” OR AB “Hyperlipidemia” OR AB “Hyperlipidemias” OR AB “Hyperlipemia” OR AB “Lipidemia” OR AB “Lipidemias” OR AB “Lipemia” OR AB “Lipemias” OR AB “Cholesterol” OR AB “Diabetes Mellitus” OR AB “Diabetes Mellitus, Type 2” OR AB “Prediabetic State” OR AB “Ketosis-Resistant Diabetes Mellitus” OR AB “Non-Insulin-Dependent Diabetes Mellitus” OR AB “Type 2 Diabetes Mellitus” OR AB “Slow-Onset Diabetes Mellitus” OR AB “Stable Diabetes Mellitus” OR AB “Maturity-Onset Diabetes Mellitus” OR AB “Maturity Onset Diabetes Mellitus” OR AB “MODY” OR AB “NIDDM” OR AB “Adult-Onset Diabetes Mellitus” OR AB “Blood Glucose” OR AB “Blood Sugar” OR AB “Hemoglobin” OR AB “Motor Activities” OR AB “Physical Activity” OR AB “Physical Activities” OR AB “Locomotor Activity” OR AB “Locomotor Activities” OR AB “Exercise” OR AB “Exercises” OR AB “Physical Exercise” OR AB “Physical Exercises” OR AB “Isometric Exercises” OR AB “Aerobic Exercise” OR AB “Aerobic Exercises” OR AB “Diet” OR AB “Nutrition” OR AB “Metabolic Syndrome” OR AB “Metabolic Cardiovascular Syndrome” OR AB “Weight Loss” OR AB “Weight Losses” OR AB “Weight Reduction” OR AB “Weight Reductions” OR AB “Body Mass Index” OR AB “BMI”)

AND

[Point and click ‘PL’ population location

(“United States” OR “US” OR “USA” OR “United States of America” OR [Alabama](http://www.ncbi.nlm.nih.gov/mesh/68000407) OR Alaska OR Arizona OR Arkansas OR California OR Colorado OR Connecticut OR Delaware OR Florida OR Georgia OR Hawaii OR Idaho OR Illinois OR Indiana OR Iowa OR Kansas OR Kentucky OR Louisiana OR Maine OR Maryland OR Massachusetts OR Michigan OR Minnesota OR Mississippi OR Missouri OR Montana OR Nebraska OR Nevada OR “New Hampshire” OR “New Jersey” OR “New Mexico” OR “New York” OR “North Carolina” OR “North Dakota” OR Ohio OR Oklahoma OR Oregon OR Pennsylvania OR “Rhode Island” OR “South Carolina” OR “South Dakota” OR Tennessee OR Texas OR Utah OR Vermont OR Virginia OR Washington OR “West Virginia” OR Wisconsin OR Wyoming)

NOT

(TI “randomized” OR TI “trial” OR TI “quality of life” OR TI “effects” OR TI “systematic review” OR TI “meta-analysis” OR TI “meta analysis”)

**SCOPUS**

(TITLE-ABS-KEY ( “access to health care” OR “accessibility of health services” OR “availability of health services” OR “health services availability” “screening rate” OR “screening rates” OR “rates of screening” OR “rate of service use” OR “rates of service use” OR “access” OR “access to care” OR “access to primary care”)

AND

TITLE-ABS-KEY (“Schizophrenia” OR “Acute Schizophrenia” OR “Catatonic Schizophrenia” OR “Paranoid Schizophrenia” OR “Process Schizophrenia” OR “Schizophreniform Disorder” OR “Schizoaffective Disorder” OR “Undifferentiated Schizophrenia” OR “Psychosis” OR “Bipolar Disorder” OR “Major Depression” OR “Neuroleptic Drugs” OR “Serious Mental Illness” OR “Serious Mental Illnesses” OR “Severe Mental Illness” OR “Severe Mental Illnesses” OR “Seriously Mentally Ill” OR “Severely Mentally Ill” OR “Serious Mental Disorder” OR “Serious Mental Disorders” OR “Severe Mental Disorder” OR “Severe Mental Disorders” OR “Paranoid Disorders” OR “Psychotic Disorders” OR “Bipolar Disorder” OR “Major Depressive Disorder” OR “Schizophrenias” OR “Schizophrenic Disorders” OR “Schizophrenic Disorder” OR “Dementia Praecox” OR “Paranoid Disorder” OR “Paranoid Psychoses” OR “Psychotic Disorder” OR “Psychosis” OR “Psychoses” OR “Schizoaffective Disorder” OR “Schizoaffective Disorders” OR “Schizophreniform Disorders” OR “Schizophreniform Disorder” OR “Brief Reactive Psychoses” OR “Brief Reactive Psychosis” OR “Bipolar Disorders” OR “Manic-Depressive Psychosis” OR “Manic Depressive Psychosis” OR “Bipolar Affective Psychosis” OR “Manic-Depressive Psychoses” OR “Mania” OR “Manias” OR “Manic State” OR “Manic States” OR “Bipolar Depression” OR “Manic Disorder” OR “Manic Disorders” OR “Major Depressive Disorders” OR “Major Depressive Disorder” OR “Involutional Psychoses” OR “Involutional Psychosis” OR “Involutional Depression” OR “Involutional Melancholia” OR “Antipsychotic” OR “Antipsyhotics” OR “Major Mental Illness” OR “Major Mental Illnesses” OR “Major Psychiatric Disorder” OR “Major Psychiatric Disorders” OR “Serious Psychiatric Disorder” OR “Serious Psychiatric Disorders” OR “Severe Psychiatric Disorder” OR “Severe Psychiatric Disorders”)

AND

TITLE-ABS-KEY ("Cardiovascular Disorders" OR "Cerebrovascular Disorders" OR "Overweight" OR "Obesity" OR "Body Mass Index" OR "Weight Loss" OR "Weight Gain" OR "Weight Control" OR "Hypertension" OR "Cholesterol" OR "Lipids" OR "Diabetes Mellitus" OR "Blood Sugar" OR "Hemoglobin" OR "Physical Activity" OR "Exercise" OR "Diets" OR "Eating Behavior" OR "Nutrition" OR "Metabolic Syndrome" OR “Cardiovascular Diseases” OR “Cardiovascular Disease” OR “Stroke” OR “Heart Disease” OR “Cardiac Diseases” OR “Vascular Disease” OR “Overweight” OR “Obese” OR “Obesity” OR “Hypertension” OR “High Blood Pressure” OR “High Blood Pressures” OR “Hyperlipidemia” OR “Hyperlipidemias” OR “Hyperlipemia” OR “Lipidemia” OR “Lipidemias” OR “Lipemia” OR “Lipemias” OR “Cholesterol” OR “Diabetes Mellitus” OR “Diabetes Mellitus, Type 2” OR “Prediabetic State” OR “Ketosis-Resistant Diabetes Mellitus” OR “Non-Insulin-Dependent Diabetes Mellitus” OR “Type 2 Diabetes Mellitus” OR “Slow-Onset Diabetes Mellitus” OR “Stable Diabetes Mellitus” OR “Maturity-Onset Diabetes Mellitus” OR “Maturity Onset Diabetes Mellitus” OR “MODY” OR “NIDDM” OR “Adult-Onset Diabetes Mellitus” OR “Blood Glucose” OR “Blood Sugar” OR “Hemoglobin” OR “Motor Activity” OR “Motor Activities” OR “Physical Activity” OR “Physical Activities” OR “Locomotor Activity” OR “Locomotor Activities” OR “Exercise” OR “Exercises” OR “Physical Exercise” OR “Physical Exercises” OR “Isometric Exercises” OR “Aerobic Exercise” OR “Aerobic Exercises” OR “Diet” OR “Nutrition” OR “Metabolic Syndrome” OR “Metabolic Cardiovascular Syndrome” OR “Weight Loss” OR “Weight Losses” OR “Weight Reduction” OR “Weight Reductions” OR “Body Mass Index” OR “BMI”)

AND

TITLE-ABS-KEY (“United States” OR “US” OR “USA” OR “United States of America” OR [Alabama](http://www.ncbi.nlm.nih.gov/mesh/68000407) OR Alaska OR Arizona OR Arkansas OR California OR Colorado OR Connecticut OR Delaware OR Florida OR Georgia OR Hawaii OR Idaho OR Illinois OR Indiana OR Iowa OR Kansas OR Kentucky OR Louisiana OR Maine OR Maryland OR Massachusetts OR Michigan OR Minnesota OR Mississippi OR Missouri OR Montana OR Nebraska OR Nevada OR “New Hampshire” OR “New Jersey” OR “New Mexico” OR “New York” OR “North Carolina” OR “North Dakota” OR Ohio OR Oklahoma OR Oregon OR Pennsylvania OR “Rhode Island” OR “South Carolina” OR “South Dakota” OR Tennessee OR Texas OR Utah OR Vermont OR Virginia OR Washington OR “West Virginia” OR Wisconsin OR Wyoming)

NOT

TITLE-ABS-KEY ( “randomized” OR “trial” OR “quality of life” OR “effects” OR “systematic review” OR “meta-analysis” OR “meta analysis”))

**EMBASE**(( ‘access to health care’:ti,ab OR ‘accessibility of health services’:ti,ab OR ‘availability of health services’:ti,ab OR ‘health services availability’:ti,ab OR ‘screening rate’:ti,ab OR ‘screening rates’:ti,ab OR ‘rates of screening’:ti,ab OR ‘rate of service use’:ti,ab OR ‘rates of service use’:ti,ab ‘access’:ti,ab OR ‘access to care’:ti,ab OR ‘access to primary care’:ti,ab) AND (‘Serious Mental Illness’:ti,ab OR ‘Serious Mental Illnesses’:ti,ab OR ‘Severe Mental Illness’:ti,ab OR ‘Severe Mental Illnesses’:ti,ab OR ‘Seriously Mentally Ill’:ti,ab OR ‘Severely Mentally Ill’:ti,ab OR ‘Serious Mental Disorder’:ti,ab OR ‘Serious Mental Disorders’:ti,ab OR ‘Severe Mental Disorder’:ti,ab OR ‘Severe Mental Disorders’:ti,ab OR ‘Schizophrenia’:ti,ab OR ‘Paranoid Disorders’:ti,ab OR ‘Psychotic Disorders’:ti,ab OR ‘Bipolar Disorder’:ti,ab OR ‘Major Depressive Disorder’:ti,ab OR ‘Schizophrenias’:ti,ab OR ‘Schizophrenic Disorders’:ti,ab OR ‘Schizophrenic Disorder’:ti,ab OR ‘Dementia Praecox’:ti,ab OR ‘Paranoid Disorder’:ti,ab OR ‘Paranoid Psychoses’:ti,ab OR ‘Psychotic Disorder’:ti,ab OR ‘Psychosis’:ti,ab OR ‘Psychoses’:ti,ab OR ‘Schizoaffective Disorder’:ti,ab OR ‘Schizoaffective Disorders’:ti,ab OR ‘Schizophreniform Disorders’:ti,ab OR ‘Schizophreniform Disorder’:ti,ab OR ‘Brief Reactive Psychoses’:ti,ab OR ‘Brief Reactive Psychosis’:ti,ab OR ‘Bipolar Disorders’:ti,ab OR ‘Manic-Depressive Psychosis’:ti,ab OR ‘Manic Depressive Psychosis’:ti,ab OR ‘Bipolar Affective Psychosis’:ti,ab OR ‘Manic-Depressive Psychoses’:ti,ab OR ‘Mania’:ti,ab OR ‘Manias’:ti,ab OR ‘Manic State’:ti,ab OR ‘Manic States’:ti,ab OR ‘Bipolar Depression’:ti,ab OR ‘Manic Disorder’:ti,ab OR ‘Manic Disorders’:ti,ab OR ‘Major Depressive Disorders’:ti,ab OR ‘Major Depressive Disorder’:ti,ab OR ‘Involutional Psychoses’:ti,ab OR ‘Involutional Psychosis’:ti,ab OR ‘Involutional Depression’:ti,ab OR ‘Involutional Melancholia’:ti,ab OR ‘Antipsychotic’:ti,ab OR ‘Antipsyhotics’:ti,ab OR ‘Major Mental Illness’:ti,ab OR ‘Major Mental Illnesses’:ti,ab OR ‘Major Psychiatric Disorder’:ti,ab OR ‘Major Psychiatric Disorders’:ti,ab OR ‘Serious Psychiatric Disorder’:ti,ab OR ‘Serious Psychiatric Disorders’:ti,ab OR ‘Severe Psychiatric Disorder’:ti,ab OR ‘Severe Psychiatric Disorders’:ti,ab) AND (‘heart disease’/de OR ‘cerebrovascular disease’/de OR ‘obesity’/de OR ‘body mass’/de OR ‘weight reduction’/de OR ‘weight gain’/de OR ‘weight control’/de OR ‘hypertension’/de OR ‘hyperlipidemia’/de OR ‘diabetes mellitus’/de OR ‘exercise’/de OR ‘diet’/de OR ‘nutrition’/de OR ‘eating habit’/de OR ‘Cardiovascular Diseases’:ti,ab OR ‘Cardiovascular Disease’:ti,ab OR ‘Stroke’:ti,ab OR ‘Heart Disease’:ti,ab OR ‘Cardiac Diseases’:ti,ab OR ‘Vascular Disease’:ti,ab OR ‘Overweight’:ti,ab OR ‘Obese’:ti,ab OR ‘Obesity’:ti,ab OR ‘Hypertension’:ti,ab OR ‘High Blood Pressure’:ti,ab OR ‘High Blood Pressures’:ti,ab OR ‘Hyperlipidemia’:ti,ab OR ‘Hyperlipidemias’:ti,ab OR ‘Hyperlipemia’:ti,ab OR ‘Lipidemia’:ti,ab OR ‘Lipidemias’:ti,ab OR ‘Lipemia’:ti,ab OR ‘Lipemias’:ti,ab OR ‘Cholesterol’:ti,ab OR ‘Diabetes Mellitus’:ti,ab OR ‘Diabetes Mellitus, Type 2’:ti,ab OR ‘Prediabetic State’:ti,ab OR ‘Ketosis-Resistant Diabetes Mellitus’:ti,ab OR ‘Non-Insulin-Dependent Diabetes Mellitus’:ti,ab OR ‘Type 2 Diabetes Mellitus’:ti,ab OR ‘Slow-Onset Diabetes Mellitus’:ti,ab OR ‘Stable Diabetes Mellitus’:ti,ab OR ‘Maturity-Onset Diabetes Mellitus’:ti,ab OR ‘Maturity Onset Diabetes Mellitus’:ti,ab OR ‘MODY’:ti,ab OR ‘NIDDM’:ti,ab OR ‘Adult-Onset Diabetes Mellitus’:ti,ab OR ‘Blood Glucose’:ti,ab OR ‘Blood Sugar’:ti,ab OR ‘Hemoglobin’:ti,ab OR ‘Motor Activity’:ti,ab OR ‘Motor Activities’:ti,ab OR ‘Physical Activity’:ti,ab OR ‘Physical Activities’:ti,ab OR ‘Locomotor Activity’:ti,ab OR ‘Locomotor Activities’:ti,ab OR ‘Exercise’:ti,ab OR ‘Exercises’:ti,ab OR ‘Physical Exercise’:ti,ab OR ‘Physical Exercises’:ti,ab OR ‘Isometric Exercises’:ti,ab OR ‘Aerobic Exercise’:ti,ab OR ‘Aerobic Exercises’:ti,ab OR ‘Diet’:ti,ab OR ‘Nutrition’:ti,ab OR ‘Metabolic Syndrome’:ti,ab OR ‘Metabolic Cardiovascular Syndrome’:ti,ab OR ‘Weight Loss’:ti,ab OR ‘Weight Losses’:ti,ab OR ‘Weight Reduction’:ti,ab OR ‘Weight Reductions’:ti,ab OR ‘Body Mass Index’:ti,ab OR ‘BMI’:ti,ab) AND (’United States’:ti,ab OR ’US’ OR ‘USA’ OR ‘United States of America’ OR [Alabama](http://www.ncbi.nlm.nih.gov/mesh/68000407) OR Alaska OR Arizona OR Arkansas OR ‘California’:ti,ab OR ‘Colorado’:ti,ab OR ‘Connecticut’:ti,ab OR ‘Delaware’:ti,ab OR ‘Florida’:ti,ab OR ‘Georgia’:ti,ab OR ‘Hawaii’:ti,ab OR ‘Idaho’:ti,ab OR ‘Illinois’:ti,ab OR ‘Indiana’:ti,ab OR ‘Iowa’:ti,ab OR ‘Kansas’:ti,ab OR ‘Kentucky’:ti,ab OR ‘Louisiana’:ti,ab OR ‘Maine’:ti,ab OR ‘Maryland’:ti,ab OR ‘Massachusetts’:ti,ab OR ‘Michigan’:ti,ab OR ‘Minnesota’:ti,ab OR ‘Mississippi’:ti,ab OR ‘Missouri’:ti,ab OR ‘Montana’:ti,ab OR ‘Nebraska’:ti,ab OR ‘Nevada’:ti,ab OR ‘New Hampshire’:ti,ab OR ‘New Jersey’:ti,ab OR ‘New Mexico’:ti,ab OR ‘New York’:ti,ab OR ‘North Carolina’:ti,ab OR ‘North Dakota’:ti,ab OR ‘Ohio’:ti,ab OR ‘Oklahoma’:ti,ab OR ‘Oregon’:ti,ab OR ‘Pennsylvania’:ti,ab OR ‘Rhode Island’:ti,ab OR ‘South Carolina’:ti,ab OR ‘South Dakota’:ti,ab OR ‘Tennessee’:ti,ab OR ‘Texas’:ti,ab OR ‘Utah’:ti,ab OR ‘Vermont’:ti,ab OR ‘Virginia’:ti,ab OR ‘Washington’:ti,ab OR ‘West Virginia’:ti,ab OR ‘Wisconsin’:ti,ab OR ‘Wyoming’:ti,ab))

**WEB OF SCIENCE**

( ‘access to health care’’ OR ‘accessibility of health services’’ OR ‘availability of health services’’ OR ‘health services availability’’ OR ‘screening rate’’ OR ‘screening rates’’ OR ‘rates of screening’’ OR ‘rate of service use’’ OR ‘rates of service use’’ ‘access’’ OR ‘access to care’’ OR ‘access to primary care’’)

AND

(“Schizophrenia” OR “Acute Schizophrenia” OR “Catatonic Schizophrenia” OR “Paranoid Schizophrenia” OR “Process Schizophrenia” OR “Schizophreniform Disorder” OR “Schizoaffective Disorder” OR “Undifferentiated Schizophrenia” OR “Psychosis” OR “Bipolar Disorder” OR “Major Depression” OR “Neuroleptic Drugs” OR “Serious Mental Illness” OR “Serious Mental Illnesses” OR “Severe Mental Illness” OR “Severe Mental Illnesses” OR “Seriously Mentally Ill” OR “Severely Mentally Ill” OR “Serious Mental Disorder” OR “Serious Mental Disorders” OR “Severe Mental Disorder” OR “Severe Mental Disorders” OR “Paranoid Disorders” OR “Psychotic Disorders” OR “Bipolar Disorder” OR “Major Depressive Disorder” OR “Schizophrenias” OR “Schizophrenic Disorders” OR “Schizophrenic Disorder” OR “Dementia Praecox” OR “Paranoid Disorder” OR “Paranoid Psychoses” OR “Psychotic Disorder” OR “Psychosis” OR “Psychoses” OR “Schizoaffective Disorder” OR “Schizoaffective Disorders” OR “Schizophreniform Disorders” OR “Schizophreniform Disorder” OR “Brief Reactive Psychoses” OR “Brief Reactive Psychosis” OR “Bipolar Disorders” OR “Manic-Depressive Psychosis” OR “Manic Depressive Psychosis” OR “Bipolar Affective Psychosis” OR “Manic-Depressive Psychoses” OR “Mania” OR “Manias” OR “Manic State” OR “Manic States” OR “Bipolar Depression” OR “Manic Disorder” OR “Manic Disorders” OR “Major Depressive Disorders” OR “Major Depressive Disorder” OR “Involutional Psychoses” OR “Involutional Psychosis” OR “Involutional Depression” OR “Involutional Melancholia” OR “Antipsychotic” OR “Antipsyhotics” OR “Major Mental Illness” OR “Major Mental Illnesses” OR “Major Psychiatric Disorder” OR “Major Psychiatric Disorders” OR “Serious Psychiatric Disorder” OR “Serious Psychiatric Disorders” OR “Severe Psychiatric Disorder” OR “Severe Psychiatric Disorders”)

AND

("Cardiovascular Disorders" OR "Cerebrovascular Disorders" OR "Overweight" OR "Obesity" OR "Body Mass Index" OR "Weight Loss" OR "Weight Gain" OR "Weight Control" OR "Hypertension" OR "Cholesterol" OR "Lipids" OR "Diabetes Mellitus" OR "Blood Sugar" OR "Hemoglobin" OR "Physical Activity" OR "Exercise" OR "Diets" OR "Eating Behavior" OR "Nutrition" OR "Metabolic Syndrome" OR “Cardiovascular Diseases” OR “Cardiovascular Disease” OR “Stroke” OR “Heart Disease” OR “Cardiac Diseases” OR “Vascular Disease” OR “Overweight” OR “Obese” OR “Obesity” OR “Hypertension” OR “High Blood Pressure” OR “High Blood Pressures” OR “Hyperlipidemia” OR “Hyperlipidemias” OR “Hyperlipemia” OR “Lipidemia” OR “Lipidemias” OR “Lipemia” OR “Lipemias” OR “Cholesterol” OR “Diabetes Mellitus” OR “Diabetes Mellitus, Type 2” OR “Prediabetic State” OR “Ketosis-Resistant Diabetes Mellitus” OR “Non-Insulin-Dependent Diabetes Mellitus” OR “Type 2 Diabetes Mellitus” OR “Slow-Onset Diabetes Mellitus” OR “Stable Diabetes Mellitus” OR “Maturity-Onset Diabetes Mellitus” OR “Maturity Onset Diabetes Mellitus” OR “MODY” OR “NIDDM” OR “Adult-Onset Diabetes Mellitus” OR “Blood Glucose” OR “Blood Sugar” OR “Hemoglobin” OR “Motor Activity” OR “Motor Activities” OR “Physical Activity” OR “Physical Activities” OR “Locomotor Activity” OR “Locomotor Activities” OR “Exercise” OR “Exercises” OR “Physical Exercise” OR “Physical Exercises” OR “Isometric Exercises” OR “Aerobic Exercise” OR “Aerobic Exercises” OR “Diet” OR “Nutrition” OR “Metabolic Syndrome” OR “Metabolic Cardiovascular Syndrome” OR “Weight Loss” OR “Weight Losses” OR “Weight Reduction” OR “Weight Reductions” OR “Body Mass Index” OR “BMI”)

AND

(“United States” OR “US” OR “USA” OR “United States of America” OR [Alabama](http://www.ncbi.nlm.nih.gov/mesh/68000407) OR Alaska OR Arizona OR Arkansas OR California OR Colorado OR Connecticut OR Delaware OR Florida OR Georgia OR Hawaii OR Idaho OR Illinois OR Indiana OR Iowa OR Kansas OR Kentucky OR Louisiana OR Maine OR Maryland OR Massachusetts OR Michigan OR Minnesota OR Mississippi OR Missouri OR Montana OR Nebraska OR Nevada OR “New Hampshire” OR “New Jersey” OR “New Mexico” OR “New York” OR “North Carolina” OR “North Dakota” OR Ohio OR Oklahoma OR Oregon OR Pennsylvania OR “Rhode Island” OR “South Carolina” OR “South Dakota” OR Tennessee OR Texas OR Utah OR Vermont OR Virginia OR Washington OR “West Virginia” OR Wisconsin OR Wyoming)
