# Supplementary material for: Screening for cardiovascular risk factors in adults with serious mental illness: a review of the evidence
Source: BMC Psychiatry. 2015 Mar 21;15:55. doi: 10.1186/s12888-015-0416-y (PMC4376086; doi:10.1186/s12888-015-0416-y)
Supplement: Additional file 2: — Search strategies. [file 12888_2015_416_MOESM2_ESM.docx]

**Appendix B: Abstraction Tool**

| **Study ID:** |
| --- |
| **Reviewer ID:** |
| **Author:** |
| **Journal:** |
| **Publication Year:** |

**Outcome of Interest (check one)**

**__Cardiovascular risk factors overall**

**__Dyslipidemia**

**__Diabetes Mellitus**

**Population Description**

| **Data source:**  **Year(s) data was collected:** |
| --- |
| **Country where study took place: USA Other­­­­­­­­­­______________________________________** |
| **Study Setting:** Hospital Mental Health Clinic Medical Clinic University CBO  Community Psychiatric Rehab Insurer Claims data across multiple settings  Other________________________ |
| **Study Population:** Community Inpatient Outpatient Other___________________________________ |

| **Detailed Description of study population**  *Describe the population in detail, e.g. note specific city/state, organization, and any other relevant clarifying details*  **Inclusion Criteria (if applicable):**  **Exclusion Criteria(if applicable):** |
| --- |

**Study Participants** *(Write “NA” for ‘not applicable’ and “CT” for ‘can’t tell’)*

**If paper presents N rather than % for race and diagnoses, write N in table (we can calculate percents from total N)*

| **Total N:** |
| --- |
| **Age Range:** |
| **Mean Age:** |
| **Percent Female:** |
| **Percent white:** |
| **Percent black:** |
| **Percent Hispanic:** |
| **Percent Other:**  *Describe other(s)_________________* |
| **Percent schizophrenia spectrum:** |
| **Percent bipolar:** |
| **Percent other psychoses:** |
| **Percent major depression:** |
| **Percent PTSD:** |
| **Percent other diagnosis:**  *Describe other(s)_________________* |

**Sampling:**

**What type of sampling does this study use (check one)?**

**__Probability**

**__Purposive**

**__Convenience**

**__Other_____________________________________________________**

**In your judgment, how representative is the sample population of the overall population?**

**__Very representative**

**__Somewhat representative**

**__Somewhat unrepresentative**

**__Very unrepresentative**

**__Can’t tell**

**Outcomes**

**Indicator (Describe)____________________________________________________**

**Units_______________________________________________________**

**Outcome Measure (e.g. 53%)___________________________________________**

**Describe Outcome Measure in Words (e.g. 53% of persons with SMI did not have access to a primary care doctor):**

**______________________________________________________________________________________________**
